# Effect of tetracycline administration on serum amylase activity in calves

**DOI:** 10.1186/2193-1801-2-330

**Published:** 2013-07-20

**Authors:** Bamdad Zendehbad, Adeleh Alipour, Hussein Zendehbad

**Affiliations:** Department of Physiology, Faculty of Specialized Veterinary Science, Research and Science Branch, Islamic Azad University, Tehran, Iran; Department of Statistic, Faculty of Sciences, Islamic Azad University-Mashhad Branch, Mashhad, Iran; Department of Biotechnology, Indian academy, centre for research & post graduate studies, Bangalore, India

**Keywords:** Amylase, Calf, Oxytetracycline

## Abstract

Tetracycline and related compounds are used extensively as broad spectrum antibiotics in the treatment of bacterial infections in ruminants. Tetracycline may cause acute pancreatitis which may result in increased serum amylase activity. However, it has been shown that administration of oxytetracycline in human results in decrease serum amylase activity. In this study changes in serum amylase activity were measured in 20 clinically healthy calves following intravenous injection of oxytetracycline hydrochloride at 10 mg/kg of body weight. Blood samples were collected at 30, 60, and 120 minutes after oxytetracycline injection. Serum amylase activity was measured using the amyloclastic assay. The activity of serum amylase was increased significantly (P < 0.05) at 30 (40.5%), 60 (35.1%), and 120 (39.3%) minutes after oxytetracycline hydrochloride administration. To the authors’ knowledge this is the first study on the acute effect of tetracycline administration on serum amylase activity in calves.

## Background

Amylase is a cytoplasmic enzyme that catalyzes the hydrolysis of complex starches (Stockham and Scott 2008). Acute pancreatitis is the most common cause of increased serum amylase activity (hyperamylasemia) and therefore, serum amylase measurement is commonly used as a diagnostic tool for the diagnosis of acute pancreatitis. Serum amylase has been in use as a diagnostic enzyme longer than any other enzymes (Hoffmann and Solter 2008).

Several isoenzymes of amylase have been identified in cattle (Gebicke-Härter and Geldermann 1977). The isoenzymes are found in a wide variety of tissues (Gebicke-Härter and Geldermann 1977). Pancreatic amylase activity in bovine is known to be very slow *in vivo* (Kay 1969; Karr et al. 1966), probably due to special digestive conditions in the ruminant; however, it is very active in the newborn calf (Siddons 1968). In contrary to human, bovine do not have salivary α-amylase (Stockham and Scott 2008). Amylase is also produced in the small intestines and liver (Hoffmann and Solter 2008). Intestinal amylase has not been shown to increase total serum amylase activity (Stockham and Scott 2008).

Tetracycline and related compounds are used extensively as broad spectrum antibiotics in the treatment of bacterial infections in ruminants. Several studies have shown that tetracycline may cause acute pancreatitis in human (Nicolau et al. 1991; Bernejee et al. 1989; Torosis and Vender 1987; Elmore and Rogge 1981; Bourke et al. 1978). It has also been suggested that oxytetracycline inhibits protein synthesis in the pancreatic exocrine (Fleischer 1976; Fleischer 1974) and specifically amylase synthesis and secretion (Tucker and Webster 1972).

Tetracyclines are commonly used in cattle; however, the effect of tetracycline administration on serum amylase activity in cattle is unknown. The purpose of this study was to assess changes in serum amylase activity following administration of oxytetracycline to clinically healthy calves.

## Materials and methods

A total of 20 clinically healthy Holstein calves (2–4 months old) were randomly selected from a commercial dairy herd in Mashhad, Iran. Health records were maintained on all calves by the dairymen and observations relating to disease were made by a production medicine veterinarian.

Blood samples were collected before administration of oxytetracycline (predose T0). Oxytetracycline hydrochloride (10%; Razak Lab, Tehran, Iran) was intravenously administered at 10 mg/kg of body weight. Then, blood samples were collected at 30, 60, and 120 min after oxytetracycline injection. Blood was drawn directly to the serum clot tube using a single jugular venepuncture and a Vacutainer needle. The samples were immediately transported to the Clinical Pathology laboratory in a cooler with ice packs and were processed within an hour of blood collection. The samples were centrifuged at 2000 *g* for 20 min at 4 C, and serum was stored at −20°C until analysis. Five samples with visual hemolysis were excluded from the study. No clinical signs of adverse effects were noted during the experiment and the calves were returned to their home pens.

Serum amylase activity was measured using the amyloclastic assay as described previously (Stockham and Scott 2008). An automated chemistry analyzer (VITALAB Selectra 2, Merck, Germany) and a commercially available kit (Pars-Azmoon Co., Iran) were used to measure amylase activity. All serum samples were tested in duplicate.

For statistical analysis, data were transferred to a Microsoft Excel spreadsheet (Microsoft Corp., Redmond, WA, USA). Using SPSS 16.0 statistical software (SPSS Inc., Chicago, IL, USA), a Pearson chi-square test and Fisher’s exact two-tailed test analysis was performed and differences were considered significant at values of P < 0.05.

The present study was approved and conducted in accordance with the recommendations of the Animal Care and Use Ethics Committee of the College of Veterinary Medicine, Islamic Azad University-Shahrekord Branch.

## Results

**Figure 1:**
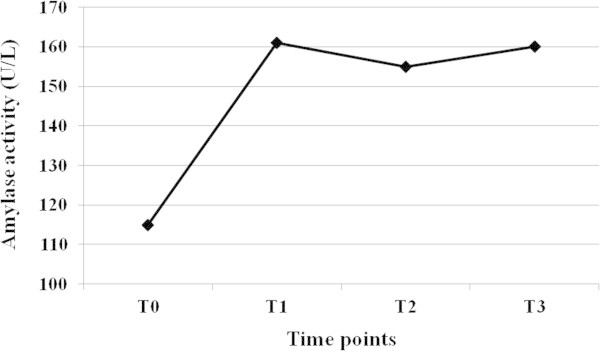
**Serum amylase activity before (T**_**0**_**) and at 30 (T**_**1**_**), 60 (T**_**2**_**), and 120 (T**_**3**_**) minutes after intravenous administration of oxytetracycline hydrochloride in 15 clinically healthy Holstein calves.**

The mean and standard deviation (SD) of serum amylase activity in 15 calves measured at 30, 60, and 120 min after oxytetracycline injection are presented in Table 1 and Figure 1. In this study, the activity of serum amylase was significantly (P < 0.05) increased in all animals at 30 (40.5%), 60 (35.1%), and 120 (39.3%) minutes after oxytetracycline hydrochloride administration. No statistically significant differences were present in serum amylase activity measured at 30, 60, and 120 min after oxytetracycline hydrochloride administration.

**Table 1:** **Serum amylase activity before and after intravenous administration of oxytetracycline hydrochloride in 15 clinically healthy Holstein calves**

Time points	Amylase activity
	(U/L)^*^
Predose (T_0_)	115.01 ± 54.03^a^
30 min post dose (T_1_)	161.56 ± 41.70^b^
60 min post dose (T_2_)	155.32 ± 42.51^b^
120 min post dose (T_3_)	160.23 ± 57.67^b^

^*^Data presented as mean ± standard deviation (SD).

^a,b^Values with no common superscript are significantly different (P < 0.05).

## Discussion

Tetracyclines are broad-spectrum antibiotics, widely used for both prevention and treatment of bacterial infections. In cattle they are also used for growth promotion. Tetracyclines have long been implicated as a causative agent in acute pancreatitis (Badalov et al. 2007; Forsmark and Baillie 2007; Nicolau et al. 1991; Torosis and Vender 1987; Elmore and Rogge 1981; Mallory and Kern 1980). Increased serum amylase activity is used as a reliable biomarker for the diagnosis of acute pancreatitis. However, several studies have shown that administration of oxytetracycline in human results in decrease serum amylase activity (Fleischer 1976). Lorenzo et al. (1999) have reported that treatment with oxytetracycline results in low amylase activity in pancreatic tissue and duodenal fluids. *In vitro* studies have shown that tetracycline has a substantial alpha amylase inhibitory activity in a dose dependent fashion (Hamdan II et al. 2004). In a recent study in rats, it has been shown that the content of amylase in pancreas significantly decreases in the animals given 50 or 200 mg kg(−1) for 21 days (Asha et al. 2007).

An amylase level more than three times above upper reference interval is highly specific for pancreatitis (Cappell 2008). In this study amylase activity was mildly to moderately increased (up to 40.5%) after administration of oxytetracycline hydrochloride. It has been shown that drug-induced pancreatitis tends to be mild and self limited (Cappell 2008). Serum amylase level increases from leakage from the inflamed pancreas into the bloodstream. Amylase is believed to rise within the first hours after the onset of pancreatitis and return to the normal range within five days (Clavien et al. 1989).

In the present study oxytetracycline hydrochloride was intravenously administered at 10 mg/kg of body weight. The half-life of elimination of oxytetracycline administered at this dosage in calves has been reported to be 6.4 ± 1.3 hour at 6 weeks of age (Burrows et al. 1987). Oxytetracycline is mostly excreted by the kidney (85-86%) and mainly through glomerular filtration (Nouws et al. 1985).

Amylase is catabolized by the kidneys and liver. Reduced amylase clearance may lead to elevation of serum amylase level, especially in cases of renal insufficiency. In the present study kidney function was not assessed; however, the animals did not show clinical signs of acute kidney failure.

## Conclusion

In conclusion, the results of this study showed that serum amylase activity was significantly (P < 0.05) increased 30 minutes after administration of tetracycline. To the authors’ knowledge this is the first study on the acute effect of tetracycline administration on serum amylase activity in calves. Further studies will be required to confirm the preliminary observations of the present study.
